# Direct and Indirect Associations of Sociodemographic Factors and Patient-Perceived Barriers With Delayed Breast Cancer Presentation: A Cross-Sectional Path Analysis

**DOI:** 10.14740/wjon2754

**Published:** 2026-06-25

**Authors:** Juan Adrian Wiranata, Susanna Hilda Hutajulu, Yufi Kartika Astari, Angelica Abigael, Mardiah Suci Hardianti, Kartika Widayati Taroeno-Hariadi, Johan Kurnianda, Yayi Suryo Prabandari, Ibnu Purwanto

**Affiliations:** a Department of Internal Medicine, Faculty of Medicine, Public Health and Nursing, Universitas Gadjah Mada, Yogyakarta, Indonesia; b Division of Hematology and Medical Oncology, Department of Internal Medicine, Faculty of Medicine, Public Health and Nursing, Universitas Gadjah Mada/Dr. Sardjito General Hospital Yogyakarta, Indonesia; c Santa Maria Hospital, Cilacap, Indonesia; d Department of Health Behaviour, Environment, and Social Medicine, Faculty of Medicine, Public Health and Nursing, Universitas Gadjah Mada, Yogyakarta, Indonesia; e Center of Health Behaviour and Promotion, Faculty of Medicine, Public Health and Nursing, Universitas Gadjah Mada, Yogyakarta, Indonesia

**Keywords:** Breast cancer, Patient-perceived barriers, Delay presentation, Path analysis

## Abstract

**Background:**

Delayed presentation remains a contributor to advanced-stage breast cancer (BC) diagnosis and poor outcomes. Although sociodemographic factors are known to influence presentation delay, patient-perceived barriers may play a role in help-seeking behavior. However, the association linking sociodemographic characteristics, patient-perceived barriers, and delayed presentation remains insufficiently understood. This study aimed to examine the direct and indirect associations between sociodemographic factors, patient-perceived barriers, and delayed BC presentation using a path analysis approach.

**Methods:**

This cross-sectional study included 150 women with BC. Sociodemographic characteristics, presentation interval, and patient-perceived barriers were collected through medical records and semi-structured interviews. Patient-perceived barriers were identified through content analysis of open-ended responses. Path analysis was conducted to estimate direct, indirect, and total effects.

**Results:**

None of the total indirect effects from sociodemographic variables to presentation delay through patient-perceived barriers were statistically significant. Five specific indirect pathways tested using the joint-significance approach were also non-statistically significant: age through fear of surgery (β = −0.002; P = 0.149), monthly income through fear of diagnosis (β = −0.029; P = 0.116), monthly income through fear of surgery (β = 0.047; P = 0.087), monthly income through preference for a female physician (β = 0.026; P = 0.135), and education through fear of surgery (β = 0.072; P = 0.051). Several sociodemographic factors showed significant direct associations with specific patient-perceived barriers. Increasing age was associated with lower fear of visiting health facilities (β = −0.245; P < 0.05) and lower fear of surgery (β = −0.149; P < 0.05). Higher income was associated with lower fear of diagnosis (β = −0.128; P < 0.05), higher fear of surgery (β = 0.170; P < 0.05), and greater preference for female physicians (β = 0.161; P < 0.05). Lower educational attainment was associated with higher fear of surgery (β = 0.283; P < 0.01), while unmarried status was associated with higher fear of healthcare costs (β = 0.383; P < 0.01) and lower likelihood of seeking complementary and alternative medicine (β = −0.130; P < 0.05). Several patient-perceived barriers were directly associated with delayed presentation, including fear of diagnosis (β = 0.165; P < 0.01), fear of surgery (β = 0.247; P < 0.01), painless symptoms (β = 0.367; P < 0.001), symptom minimization (β = 0.105; P < 0.05), perceived busyness (β = 0.244; P < 0.01), and preference for female physicians (β = 0.105; P < 0.05).

**Conclusion:**

Although the hypothesized mediation of sociodemographic factors by patient-perceived barriers was not supported, several sociodemographic factors were associated with distinct barrier profiles, and several patient-perceived barriers were associated with delayed presentation. These findings highlight the importance of addressing modifiable barriers to promote earlier presentation and improve BC outcomes.

## Introduction

Breast cancer (BC) is one of the most common malignancies in women worldwide. In Indonesia, BC accounts for 30.9% of women’s cancer cases and 11% of cancer deaths in 2020 [1]. In Yogyakarta Province, BC affects mostly younger women and often presents at advanced stages [2]. A local study reported a 5-year overall survival rate of 51% [3], lower than in nearby Asian countries.

Delays in presentation can impede early identification and diagnosis of BC, which in turn increases the burden of advanced-stage disease at initial presentation and ultimately results in poorer prognosis and reduced survival. Numerous studies found that factors such as lower education, older age, lower socioeconomic status, rural residence, and limited healthcare access are associated with delay in presentation [2, 4, 5]. Consequently, these delays lead to worse outcomes and add considerable constraints to the healthcare system, which must then provide more complex, intensive treatment.

Presentation delay might also be caused by factors beyond sociodemographic and clinical determinants, as evidence suggests that patient-perceived barriers play a pivotal mediating role in shaping help-seeking behavior. Barriers include the perception of a painless symptom, the attribution of symptoms to a non-serious condition (symptom minimization), fear of cancer diagnosis, and fear of surgical procedures [2, 4]. All are observed to significantly affect cognitive appraisal and emotional processing, potentially leading to delayed recognition of symptom seriousness and delayed medical consultation. Understanding these internal psychological and cognitive barriers is essential for developing targeted interventions that encourage timely presentation and improve BC outcomes.

One method that might offer a valuable methodological approach for examining the role of patient-perceived barriers as mediators between sociodemographic factors and presentation delay is path analysis. Path analysis allows for the simultaneous estimation of direct, indirect, and total effects among multiple variables within a hypothesized causal framework. Path analysis might help assess whether sociodemographic characteristics indirectly influence delays through their association with patient-perceived barriers, providing insight into complex relationships among determinants and enabling distinction of the contributions of mediating variables. Moreover, this analysis is well-suited for testing theoretical models of health behavior, thereby helping clarify the associations underlying delays in presentation [6].

This study aimed to examine the direct and indirect associations between sociodemographic factors, patient-perceived barriers as reasons for delay, and delayed BC presentation through path analysis, which may provide an understanding of how these factors interact among BC patients and identify possible actionable targets for intervention that could promote earlier presentation and improve outcomes.

## Materials and Methods

### Study participants and data collection

Subjects were retrospectively recruited from a primary study that aimed to analyze chemotherapy-related toxicity and its impact on survival in 250 patients with BC. The study was conducted at the Hematology and Medical Oncology Division, “Tulip”/Integrated Cancer Clinic, Dr. Sardjito General Hospital, Yogyakarta, Indonesia, between July 2018 and March 2022. Eligible subjects were women aged 18 or older with histopathologically confirmed BC and an Eastern Cooperative Oncology Group (ECOG) performance status of 0 or 1. Subjects with terminal illness, severe comorbidities, or those unable to provide accurate information due to psychotic disorders or any other causes were excluded to mitigate potential limitations in recall and interview coherence, as well as to avoid causing undue distress to these vulnerable groups.

A semi-structured questionnaire was developed to collect sociodemographic data, presentation delay, and reasons for delay, as described in detail in the previous study [2]. During development, three independent general practitioners performed forward and backward translations into Bahasa Indonesia. The questionnaire was then assessed for face validity through a pilot test with six individuals without a medical background. These participants were convenience sampled via contacts known to the research team. Trained research team members conducted face-to-face interviews from September 2020 to February 2021. Seventy-seven interviews occurred at the clinic. Due to the COVID-19 pandemic and travel restrictions, 74 interviews took place by phone. The study received ethical approval from the Faculty of Medicine, Public Health, and Nursing of Universitas Gadjah Mada/Dr. Sardjito General Hospital Joint Ethics Committee (Reference Number: KE/FK/0444/EC/2020). The study was conducted in compliance with the ethical standards of the responsible institution on human subjects as well as with the Helsinki Declaration.

### Study variables

Participant age was defined at BC diagnosis. Monthly income included individual or household earnings and was classified as ≤ 3 million Rupiah or > 3 million Rupiah (referred to as an income cut-off for happiness index > 70 by the Indonesian Ministry of National Development Planning in 2021 [7]). Educational attainment was measured as the highest level of formal education completed, categorized as senior high school or higher (including a bachelor’s degree or higher), and no formal education or only primary or junior high school. Marital status was dichotomized as married or single, widowed, and divorced.

The presentation duration interval was defined as the time from initial BC symptom onset to the patient’s first visit to a health facility or medical professional (doctor, nurse, or midwife). During the interview, subjects were asked about the date of symptom onset and the date of their first consultation. If participants could not recall exact dates, uncertain dates were handled using a procedure adapted from the Cancer Symptom Interval Measure (C-SIM) protocol for calculating “pseudo-exact” dates from estimated dates. First, participants were encouraged to estimate the period in months. Interviewers then helped refine these estimates by linking them to notable occasions or holidays to narrow the date range. If only a month was given, follow-up questions aimed to find the exact day or relate it to events within that month. Documented surgery dates in clinical records were used as benchmarks, as these were often key milestones for patients. When exact dates were unknown, participants were asked to provide a month or a month range, along with the year. If only a month was provided, the 15th was used as the estimated date; for a range of months, the midpoint between the 15th of those months was used. If only the year were available, June 30 was used as the estimated date [3, 4, 8].

Presentation delay was then determined based on the presentation duration interval, with intervals of more than 3 months considered delayed presentations, in accordance with previous literature [3, 4]. To address patient-perceived barriers, open-ended questions were included to elicit subjects’ reasons for delaying presentation.

### Statistical analysis

A content analysis of the interview transcripts was conducted to assess patient-perceived barriers, based on participants’ open-ended responses to the question of delayed presentation [9]. Two research team members independently generated inductive codes without a predetermined framework. Codes were compared, collated, and refined into candidate themes representing patient-perceived barriers. Discrepancies were resolved by consensus with consultation with a third research team member. Themes were then defined and named accordingly. The theme with only one response was excluded because it captured only prominent and recurring patterns of patient-perceived barriers [9, 10]. Descriptive analysis was performed to observe the distribution of subjects’ characteristics. Subject characteristics were summarized using mean and standard deviation (SD), median, or frequencies as appropriate.

**Figure 1:**
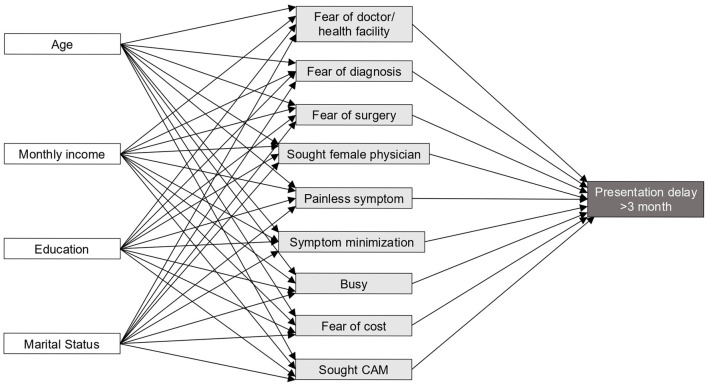
Hypothesized pathways between sociodemographic variables and presentation delay. White boxes represent sociodemographic variables, light gray boxes represent patient-perceived barrier mediators, and the dark gray box represents presentation delay of more than 3 months.

Path analysis was conducted using structural equation modeling with maximum likelihood estimation to examine the hypothesized causal pathway linking sociodemographic variables to presentation delay, mediated by patient-perceived barriers (Fig. 1). Path analysis is a subset of structural equation modeling that enables the estimation of regression coefficients corresponding to the direct, indirect, and total effects among variables. It is beneficial for testing theoretical models that propose causal relationships between a set of observed variables. The beta estimates in our path analysis represent standardized regression coefficients and are interpreted similarly to beta estimates obtained from generalized linear models, indicating the expected change in a dependent variable for a one-unit change in the independent variable [6]. To explore the possibility of pathway-specific mediation, following the joint-significance approach to mediation testing [11], specific indirect effects were estimated for the subset of candidate pathways in which both constituent direct paths were statistically significant.

A P-value of < 0.05 was considered statistically significant. Model fit was evaluated using the root mean square error of approximation (RMSEA), standardized root mean square residual (SRMR), Comparative Fit Index (CFI), and Tucker–Lewis Index (TLI) [6]. Bootstrapped standard errors and corresponding 95% confidence intervals (CIs) were computed using 1,000 replicates. All statistical analyses were carried out using STATA software version 17 (StataCorp, College Station, TX).

## Results

### Study participants

A total of 214 patients had been registered in the main study. Of these, three dropped out, and 38 had died. Among the 173 eligible patients, 16 did not respond to the invitation, and six declined to participate. One patient was excluded due to an inability to recall information or communicate effectively during the interview. In total, 150 patients were included in the final analysis.

The mean age of the participants was 53.2 years. The majority of participants were aged ≤ 60 years (80.7%), reported a monthly income of less than 3 million IDR (70.0%), attained formal education from senior high school to higher degree (57.3%), were married (83.3%), and experienced a presentation delay of ≤ 3 months (55.3%). Reasons for delay as patient-perceived barriers identified in the analysis were painless symptom (28.7%; attributed their delay in seeking care to symptoms experienced not associated with any pain or physical discomfort) and symptom minimization (22.7%; the attribution of symptoms to a non-serious condition), followed by fear of surgery (15.3%), perceived busyness (11.3%), fear of doctor or health facility visit (4.7%), sought complementary and alternative medicines (CAM) (4.0%), fear of diagnosis (4.0%), fear of cost (2.7%), and sought female physician (Table 1).

**Table 1:** Study Characteristics (N = 150)

Variables	Frequency (%)
Age (mean ± SD)	53.2 ± 9.0
≤ 60 years	121 (80.7)
> 60 years	29 (19.3)
Monthly income	
< 3 million Rupiah	105 (70)
> 3 million Rupiah	45 (30)
Educational attainment	
Senior high school to higher degree	86 (57.3)
No formal education to junior high school	64 (42.7)
Marital status	
Married	125 (83.3)
Single/widowed/divorced	25 (16.7)
Presentation interval (median, min–max, in days)	61 (0–8,378)
Presentation delay	
≤ 3 months	83 (55.3)
> 3 months	67 (44.7)
Patient-perceived barriers	
Sought female physician	2 (1.3)
Fear of cost	4 (2.7)
Sought CAM	6 (4.0)
Fear of diagnosis	6 (4.0)
Fear of doctor/health facility visit	7 (4.7)
Perceived busyness	17 (11.3)
Fear of surgery	23 (15.3)
Symptom minimization	34 (22.7)
Painless symptom	43 (28.7)

CAM: complementary and alternative medicines; max: maximum; min: minimum; SD: standard deviation.

### Path estimates and mediation

**Figure 2:**
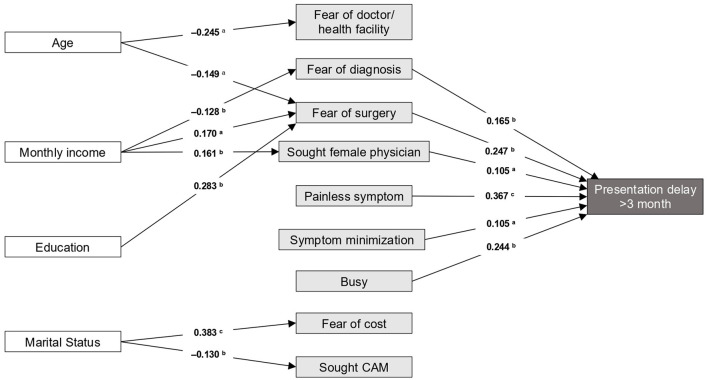
Path analysis for the relationship sociodemographic variables and presentation delay. ^a^P < 0.05; ^b^P < 0.01; ^c^P < 0.001.

Our model depicting the relationships among sociodemographic variables, patient-perceived barriers, and presentation delay is shown in Figure 2. The model fit indices of RMSEA of 0.064 and SRMR of 0.063 suggest an acceptable fit. However, the indices of CFI of 0.732 and TLI of 0.430 were under the threshold for adequate fit. The direct, total indirect, and specific indirect effects of sociodemographic variables on presentation delay of more than 3 months, mediated by patient-perceived barriers, are presented in Supplementary Material 1 (wjon.elmerpub.com).

None of the total indirect effects from the four sociodemographic variables to presentation delay reached statistical significance: age (β = −0.003, 95% CI −0.008, 0.003), monthly income (β = 0.145, 95% CI −0.087, 0.116), educational attainment (β = 0.066, 95% CI −0.041, 0.173), and marital status (β = 0.083, 95% CI −0.055, 0.221). The estimation of specific indirect effects using the joint-significance approach revealed that none of the five specific indirect pathways reached statistical significance: age through fear of surgery (β = −0.002, 95% CI −0.005, 0.001), monthly income through fear of diagnosis (β = −0.029, 95% CI −0.066, 0.007), monthly income through fear of surgery (β = 0.047, 95% CI −0.007, 0.100), monthly income through preference for a female physician (β = 0.026, 95% CI –0.008, 0.060), and education through fear of surgery (β = 0.072, 95% CI −0.001, 0.145) (Supplementary Material 1, wjon.elmerpub.com). In the structural model, several direct associations between sociodemographic characteristics and patient-perceived barriers reached statistical significance. Increased age was associated with lower fear of doctor or health-facility visits (β = −0.245, 95% CI −0.418, −0.071) and lower fear of surgery (β = −0.149, 95% CI −0.294, −0.003). Higher income (> 3 million Rupiah) was associated with lower fear of diagnosis (β = −0.128, 95% CI −0.225, −0.032), higher fear of surgery (β = 0.170, 95% CI 0.005, 0.334), and a greater likelihood of reporting preference for a female physician as a barrier (β = 0.161, 95% CI 0.046, 0.277). Lower educational attainment (no formal education to junior high school) was associated with higher fear of surgery (β = 0.283, 95% CI 0.104, 0.462). Being single, widowed, or divorced was associated with higher fear of cost (β = 0.383, 95% CI 0.184, 0.582) and lower reporting of CAM use as a first option (β = −0.130, 95% CI −0.224, −0.035). Six patient-perceived barriers were directly associated with presentation delay of more than 3 months: fear of diagnosis (β = 0.165, 95% CI 0.065, 0.264), fear of surgery (β = 0.247, 95% CI 0.099, 0.397), preference for a female physician (β = 0.105, 95% CI 0.020, 0.189), painless symptom (β = 0.367, 95% CI 0.223, 0.511), symptom minimization (β = 0.105, 95% CI 0.020, 0.189), and perceived busyness (β = 0.244, 95% CI 0.094, 0.394) (Supplementary Material 1, wjon.elmerpub.com).

## Discussion

Growing evidence supports a strong association between delays in presentation, stage at diagnosis, and subsequent patient survival. Prolonged presentation intervals increase the likelihood of cancer being detected at an advanced stage, which is associated with poorer prognosis and reduced quality of life [2, 4]. Presentation delay reflects the interplay of multiple levels of determinants shaping health behavior. The interconnected layers of individual cognitive and emotional factors (awareness, health beliefs, emotional responses), social-environmental factors (work commitments, peer support, supportive networks), contextual factors (employment, income, education, healthcare access), and macro-level cultural and economic conditions collectively contribute to delays in presentation [12].

In our analysis, we found that the mediation model was not supported and that the findings are best interpreted as describing two sets of direct associations within a structural model. We have explored factors across these multiple layers and their interactions to better understand the complex thought processes underlying delayed presentation. Several sociodemographic variables were significantly associated with specific patient-perceived barriers. Multiple barriers also showed strong direct associations with delayed presentation. However, these perceived barriers did not significantly mediate the relationship between sociodemographic factors and presentation delay. This finding indicates that sociodemographic influences on delayed presentation do not operate through a single dominant mediating pathway, even when individual structural paths are significant. The individual effects of these barriers are insufficient in magnitude or consistency to produce statistically detectable mediation. Previous work has shown that statistical tests of indirect effects can have low power under realistic sample size and effect size conditions, making mediation difficult to detect even when structural paths exist [13]. The results also highlight that patient-perceived barriers are associated determinants of delayed presentation.

The overall model fit was mixed. Although the RMSEA and SRMR fell within commonly accepted thresholds, the CFI (0.732) and TLI (0.430) fell well below the conventional cutoffs. Several factors may contribute to this divergence, including low base rates in several patient-perceived barriers (e.g., “sought female physician” n = 2; “fear of cost” n = 4), which might produce sparse cross-classifications that disproportionately depress incremental fit. Furthermore, some relationships among barriers may be non-linear or moderated rather than additive, a possibility that the present specification does not capture. Accordingly, individual structural parameters can be interpreted as descriptive associations within the specified model, but the overall fit leaves substantive covariance unexplained and indicates a need for refinement in larger studies [14].

### Sociodemographic factors and their influence on perceived barriers

In this study, older age was associated with lower fear of visiting health facilities and lower fear of surgery. The first finding contrasts with a previous report that 22.5% of adults avoided medical care due to examination-related discomfort and fear of serious illness [15], with these patterns modulated by health literacy [16]. In addition, older age was significantly associated with lower fear of surgery, consistent with prior evidence demonstrating a negative correlation between fear of surgery and age [17], plausibly reflecting the higher preoperative anxiety observed in younger patients [18]. These age-related differences may collectively reflect greater familiarity with healthcare settings, better emotional regulation, increased acceptance of medical interventions, and a greater readiness to confront health-related risks with advancing age [17, 18].

Higher income was linked to lower fear of cancer diagnosis, consistent with evidence that higher income reduces health anxiety [19] and less avoidance of serious illness [20]. Unexpectedly, higher income was also associated with greater fear of surgery, contrasting prior reports of greater preoperative anxiety among lower-income individuals due to financial concerns [21–23], which may reflect heightened expectations for treatment quality and outcomes among higher-income patients [24]. Higher-income patients also more frequently preferred female physicians, consistent with prior studies of provider gender preference [21–23], potentially reflecting the perception of female physicians as more empathetic [25], greater comfort during examination of intimate organs [25, 26], and the reinforcing influence of cultural norms in more conservative societies [26].

Lower educational attainment was associated with greater fear of surgery, consistent with evidence that limited health literacy elevates preoperative anxiety [27, 28], and that lower education correlates with anxiety in medical and surgical settings [18, 21, 29]. Higher education may attenuate this anxiety through cognitive and informational resources—critical thinking, access to health information, and understanding of procedures [18]. Being single, widowed, or divorced was associated with greater fear of healthcare costs and lower reporting of CAM as a barrier. The heightened cost-related fear plausibly reflects the financial vulnerability of unmarried women within reduced household financial support [30, 31], while the lower CAM reporting is consistent with evidence that married individuals are more likely to use CAM, possibly because of the shared decision-making and mutual support that marriage facilitates [32–34].

### Patient-perceived barriers as factors associated with delayed presentation

Several patient-perceived barriers were associated with delayed presentation in the model. Among the most prominent barriers was fear of a cancer diagnosis, which is a known psychological reason that makes people delay seeking help. Finding from previous study shows that 34% of women waited to get care because they were afraid of being told that they had BC [35]. This fear may come from worries about dying after a cancer diagnosis, which can make people avoid care or put off seeking care [36].

Fear of surgery was found to be a substantial barrier, particularly in the context of BC. Fears of diminished sexuality, loss of femininity, or altered self-worth related to breast surgery might delay patients’ presentation [37, 38]. Evidence suggests that preoperative psychosocial screening and supportive interventions may help reduce these fears [39]. Cognitive barriers, such as perceiving painless symptoms and symptom minimization, were frequently reported. Patients may misinterpret painless breast lumps as benign or self-limiting and delay medical presentation, as patients assume that symptoms will resolve [5, 38]. This highlights persistent gaps in public awareness of BC symptoms and underscores the need for targeted educational initiatives [40, 41].

In addition, practical and culturally specific barriers were significantly associated with delayed presentation, with perceived busyness serving as a key factor. This sense of busyness, especially due to competing work and family responsibilities, often leads individuals to deprioritize their health [42]. This calls attention to the need for more flexible healthcare delivery models, such as extended clinic hours and mobile health services [43].

Finally, preference for female physicians emerged as a key barrier for some participants, as women might delay or avoid medical consultations when female healthcare providers are unavailable. This is particularly pronounced in Indonesian women, where cultural and religious norms strongly influence healthcare-seeking behaviors [44–46]. Addressing this barrier requires system-level responses. Improving the availability of female providers and integrating culturally competent care that respects patient preferences, beliefs, and religious practices can increase trust, patient satisfaction, and timely access to care [47, 48].

### Strength and limitations

This study applies a theory-driven path analysis to examine complex relationships between sociodemographic characteristics, patient-perceived barriers, and delayed BC presentation, enabling a nuanced understanding of how distal and proximal factors might interact. By incorporating a broad range of patient-perceived barriers across cognitive, emotional, and cultural dimensions, the analysis moves beyond isolated factors to capture the multifaceted nature of help-seeking behavior, thereby providing empirically grounded insights to inform targeted interventions that address modifiable patient-level barriers alongside sociodemographic context.

Several limitations should be acknowledged. This study was conducted at a single center, which may limit generalizability, and further multicenter studies are required to confirm these findings. The modest sample size in this study, relative to the complexity of the path model, limited statistical power for detecting indirect effects, which is a recognized challenge in mediation analysis when constituent paths are of small-to-moderate magnitude [13]. Larger multicenter studies are warranted to further confirm the absence of mediation in the hypothesized model.

Additionally, although efforts to anchor recall to events or dates have been made through the use of recall protocols [8], self-reported data on timing and intervals, and reasons for delay as patient-perceived barriers, may still be affected by recall bias and recall uncertainty cannot be fully eliminated. Recall could also be selectively distorted by the emotional salience of the subsequent cancer diagnosis or surgery, producing either “compression” or “telescoping” of recalled intervals [49]. Moreover, since patient-perceived barriers were assessed retrospectively, some reported barriers may represent post hoc rationalizations [50].

Patient-perceived barriers were assessed using single-item categorical responses derived from open-ended interview content rather than validated psychometric instruments. Multidimensional constructs (e.g., fear of diagnosis or fear of surgery) are represented as binary indicators that capture the presence of the concern as a stated reason for delay but not its intensity, duration, or specific cognitive content. Additionally, since themes mentioned by only a single participant were excluded in order to avoid unstable parameters, the exclusion may have removed some idiosyncratic but potentially meaningful barriers. Future qualitative studies with larger samples would be better positioned to examine these lower-frequency responses.

Because the data are cross-sectional and barriers were assessed retrospectively, temporal ordering between barriers and presentation delay was not established, and the path-analytic findings should therefore be interpreted as exploratory associations within a hypothesized structural model rather than as evidence of causal pathways. In addition, the presumed directionality between perceived barriers and presentation delay may be bidirectional, a relationship that has not been evaluated in this study.

### Conclusions

This study shows that patient-perceived barriers are associated with delayed BC presentation. While sociodemographic factors were associated with specific barriers, these barriers did not significantly mediate the association between sociodemographic characteristics and presentation delay, which is shown in the non-significant indirect effect in the hypothesized model. These suggest that sociodemographic influences might not act through a single dominant pathway. Rather, the findings indicate that patient-perceived barriers operate as factors associated with delayed presentation within the modelled framework of delayed presentation across different sociodemographic groups. Addressing these modifiable cognitive, emotional, practical, and cultural barriers may therefore be important for promoting earlier presentation and improving BC outcomes.

## Supplementary Material

Suppl 1 Direct and indirect effects of sociodemographic factors and patient-perceived barriers on delayed breast cancer presentation.

## Data Availability

Any inquiries regarding supporting data availability of this study should be directed to the corresponding author.

## Abbreviations

BC: breast cancer
CAM: complementary and alternative medicines
CFI: Comparative Fit Index
CI: confidence interval
ECOG: Eastern Cooperative Oncology Group
RMSEA: root mean square error of approximation
SRMR: standardized root mean square residual
TLI: Tucker–Lewis Index
