# Supplementary material for: Direct and Indirect Associations of Sociodemographic Factors and Patient-Perceived Barriers With Delayed Breast Cancer Presentation: A Cross-Sectional Path Analysis
Source: World J Oncol. 2026 Jun 25;17(4):454–62. doi: 10.14740/wjon2754 (PMC13375438; doi:10.14740/wjon2754)
Supplement: Suppl 1 — Direct and indirect effects of sociodemographic factors and patient-perceived barriers on delayed breast cancer presentation. [file wjon-17-04-454-s001.docx]

**Suppl 1**. Direct and Indirect Effects of Sociodemographic Factors and Patient-perceived Barriers on Delayed Breast Cancer Presentation

|  | | | | | | |
| --- | --- | --- | --- | --- | --- | --- |
| **Variables** | | | **Direct Effect** | | **Indirect Effect** | |
| **Independent Variable** | **Mediator Variable** | **Dependent Variable** | **Beta**  **(95% CI)** | **p-value** | **Beta (95% CI)** | **p-value** |
| Age |  | Fear of visit | –0.006  (–0.011; –0.001) | **0.029*** |  | |
| Monthly income |  |  | 0.023  (–0.060; 0.106) | 0.587 |  |  |
| Education |  |  | 0.001  (–0.059; 0.062) | 0.963 |  |  |
| Marital Status |  |  | –0.007  (–0.057; 0.041) | 0.762 |  |  |
| Age |  | Fear of diagnosis | 0.001  (–0.002; 0.003) | 0.566 |  |  |
| Monthly income |  |  | –0.055  (–0.108; –0.001) | **0.045*** |  |  |
| Education |  |  | –0.017  (–0.095; 0.061) | 0.675 |  |  |
| Marital Status |  |  | 0.083  (–0.047; 0.213) | 0.201 |  |  |
| Age |  | Fear of surgery | –0.006  (–0.012; 0.0001) | 0.054 |  |  |
| Monthly income |  |  | 0.134  (0.002; 0.265) | **0.049*** |  |  |
| Education |  |  | 0.206  (0.068; 0.344) | **0.004*** |  |  |
| Marital Status |  |  | 0.102  (–0.060; 0.264) | 0.215 |  |  |
| Age |  | Sought female physician | –0.001  (–0.002; 0.002) | 0.939 |  |  |
| Monthly income |  |  | 0.040  (–0.009; 0.090) | 0.112 |  |  |
| Education |  |  | –0.007  (–0.021; 0.007) | 0.344 |  |  |
| Marital Status |  |  | –0.008  (–0.030; 0.015) | 0.500 |  |  |
| Age |  | No pain | –0.001  (–0.003; 0.002) | 0.983 |  |  |
| Monthly income |  |  | –0.138  (–0.319; 0.042) | 0.136 |  |  |
| Education |  |  | –0.037  (–0.213; 0.139) | 0.678 |  |  |
| Marital Status |  |  | 0.020  (–0.200; 0.241) | 0.856 |  |  |
| Age |  | Symptom Minimization | –0.004  (–0.013; 0.004) | 0.286 |  |  |
| Monthly income |  |  | 0.007  (–0.158; 0.173) | 0.930 |  |  |
| Education |  |  | 0.028  (–0.135; 0.192) | 0.737 |  |  |
| Marital Status |  |  | 0.049  (–0.143; 0.241) | 0.618 |  |  |
| Age |  | Perceived busyness | 0.005  (–0.001; 0.011) | 0.094 |  |  |
| Monthly income |  |  | 0.040  (–0.083; 0.163) | 0.526 |  |  |
| Education |  |  | 0.021  (–0.074; 0.116) | 0.666 |  |  |
| Marital Status |  |  | –0.026  (–0.179; 0.125) | 0.733 |  |  |
| Age |  | Fear of cost ^b^ | –0.001  (–0.003; 0.002) | 0.734 |  |  |
| Monthly income |  |  | 0.014  (–0.060; 0.087) | 0.717 |  |  |
| Education |  |  | 0.004  (–0.066; 0.074) | 0.904 |  |  |
| Marital Status |  |  | 0.166  (0.019; 0.312) | **0.027*** |  |  |
| Age |  | Sought CAM | 0.002  (–0.002; 0.006) | 0.410 |  |  |
| Monthly income |  |  | –0.017  (–0.082; 0.047) | 0.600 |  |  |
| Education |  |  | 0.031  (–0.038; 0.100) | 0.377 |  |  |
| Marital Status |  |  | –0.068  (–0.134; –0.002) | **0.043*** |  |  |
| Fear of visit |  | Presentation delay >3 months | 0.306  (–0.130; 0.742) | 0.169 |  |  |
| Fear of diagnosis |  |  | 0.584  (0.365; 0.803) | **<0.001*** |  |  |
| Fear of surgery |  |  | 0.372  (0.135; 0.609) | **0.002*** |  |  |
| Sought female physician |  |  | 0.544  (0.307; 0.781) | **<0.001*** |  |  |
| No pain |  |  | 0.326  (0.141; 0.512) | **0.001*** |  |  |
| Symptom minimization |  |  | 0.209  (0.007; 0.411) | **0.043** |  |  |
| Perceived busyness |  |  | 0.317  (0.068; 0.566) | **0.013*** |  |  |
| Fear of cost |  |  | –0.009  (–0.337; 0.320) | 0.958 |  |  |
| Sought CAM |  |  | 0.058  (–0.252; 0.369) | 0.712 |  |  |
| Age ^a^ | All patient-perceived barriers | Presentation delay >3 months |  | | –0.003  (–0.008; 0.003) | 0.328 |
| Monthly income ^a^ |  |  |  |  | 0.145  (–0.087; 0.116) | 0.779 |
| Education ^a^ |  |  |  |  | 0.066  (–0.041; 0.173) | 0.229 |
| Marital Status ^a^ |  |  |  |  | 0.083  (–0.055; 0.221) | 0.239 |
| Age ^b^ | Fear of surgery | Presentation delay >3 months |  |  | –0.002  (–0.005; 0.001) | 0.149 |
| Monthly income ^b^ | Fear of diagnosis |  |  |  | –0.029  (–0.066; 0.007) | 0.116 |
| Monthly income ^b^ | Fear of surgery |  |  |  | 0.047  (–0.007; 0.100) | 0.087 |
| Monthly income ^b^ | Sought female physician |  |  |  | 0.026  (–0.008; 0.060) | 0.135 |
| Education ^b^ | Fear of surgery |  |  |  | 0.072  (–0.001; 0.145) | 0.051 |
| *P < 0.05  ^a^Represents total indirect effect  ^b^Specific indirect effect estimated for the subset of candidate pathways in which both constituent direct paths were statistically significant following the joint-significance approach to mediation testing.  Abbreviations: CI: confidence interval; CAM: complementary alternative medicine | | | | | | |
